# The SNARE protein Vti1b is recruited to the sites of BCR activation but is redundant for antigen internalisation, processing and presentation

**DOI:** 10.3389/fcell.2022.987148

**Published:** 2022-08-30

**Authors:** Amna Music, Blanca Tejeda-González, Diogo M. Cunha, Gabriele Fischer von Mollard, Sara Hernández-Pérez, Pieta K. Mattila

**Affiliations:** ^1^ Institute of Biomedicine, and MediCity Research Laboratories, University of Turku, Turku, Finland; ^2^ Turku Bioscience, University of Turku and Åbo Akademi University, Turku, Finland; ^3^ InFLAMES Research Flagship Center, University of Turku, Turku, Finland; ^4^ Fakultät für Chemie, Biochemie III, Universität Bielefeld, Bielefeld, Germany

**Keywords:** adaptive immunology, B cells, vesicular traffcking, BCR-B cell receptor, signalling, SNARE (soluble N-ethylmaleimide-sensitive fusion protein attachment protein receptor), VTI1B, immune synapse

## Abstract

In order to fulfil the special requirements of antigen-specific activation and communication with other immune cells, B lymphocytes require finely regulated endosomal vesicle trafficking. How the endosomal machinery is regulated in B cells remains largely unexplored. In our previous proximity proteomic screen, we identified the SNARE protein Vti1b as one of the strongest candidates getting accumulated to the sites of early BCR activation. In this report, we follow up on this finding and investigate the localisation and function of Vti1b in B cells. We found that GFP-fused Vti1b was concentrated at the Golgi complex, around the MTOC, as well as in the Rab7^+^ lysosomal vesicles in the cell periphery. Upon BCR activation with soluble antigen, Vti1b showed partial localization to the internalized antigen vesicles, especially in the periphery of the cell. Moreover, upon BCR activation using surface-bound antigen, Vti1b polarised to the immunological synapse, colocalising with the Golgi complex, and with lysosomes at actin foci. To test for a functional role of Vti1b in early B cell activation, we used primary B cells isolated from Vit1b-deficient mouse. However, we found no functional defects in BCR signalling, immunological synapse formation, or processing and presentation of the internalized antigen, suggesting that the loss of Vti1b in B cells could be compensated by its close homologue Vti1a or other SNAREs.

## Introduction

B lymphocytes, together with T lymphocytes, form the adaptive immune system. After specific antigen recognition *via* the B cell receptor (BCR) and the subsequent activation, B cells ultimately differentiate into antibody-secreting plasma cells and generate humoral responses against pathogens. Antigen binding to the BCR is a robust and multi-branched trigger that leads not only to BCR signalling and transcriptional changes, but also to a plethora of other cellular processes. For instance, BCR activation leads to cytoskeleton reorganisation, cell polarisation, endocytosis, and vesicle traffic (Harwood and Batista, 2010b; Kuokkanen et al., 2015)*.* Coordinated engagement and regulation of such a wide variety of interlinked pathways still challenges our understanding of the early events of B cell activation. Although all cells need a tight regulation of the endosomal traffic, this is imperative in immune cells, as they rely on the endosomal traffic to regulate events such as signal transduction, phagocytosis, degranulation, cytokine secretion or antigen presentation. Thus, to no surprise, vesicular proteins such as Rab GTPases, soluble N-ethylmaleimide-sensitive factor attachment protein receptor (SNARE) proteins, and their effectors play a role in the immune responses and pathogen invasion (Pei et al., 2012; Krzewski and Cullinane, 2013; Prashar et al., 2017; Spanò and Galán, 2018).

In our previous study, we set up a proteomic approach using APEX2, a proximity labelling enzyme, to unveil a plethora of proteins recruited towards the BCR signalling sites upon antigen encounter (Awoniyi et al., 2020). We identified with high confidence 1,677 recruited proteins and analysed their dynamics following BCR activation. In addition to numerous BCR signalling proteins, we detected proteins linked to cytoskeleton remodelling, endocytosis and vesicle traffic. One of the hits we identified in this dataset was the SNARE protein Vti1b (vesicle transport through interaction with t-SNAREs homolog 1B *or* VPS10 (Vacuole Protein Sorting 10)-interacting protein), which showed the strongest intensity enrichment (Log_2_ fold-change of 5) at 15 min. The SNARE superfamily consists of more than 30 SNARE proteins in mammals and they play a central role in membrane fusion. Depending on a central residue in the SNARE complex, SNAREs can be classified as arginine-containing SNAREs (R-SNAREs) and glutamine-containing SNAREs (Q-SNAREs; Qa, Qb, Qc). Vti1 (VTI gene) belongs to the Q-SNARE subclass and two different homologs, Vti1a and Vti1b, are found in mammals. Vti1a/b regulate endo-lysosomal trafficking (Advani et al., 1998; Kreykenbohm et al., 2002; Pryor et al., 2004; Emperador-Melero et al., 2018) and their loss leads to lethal impairments in neuronal development (Kunwar et al., 2011). Vti1b has been mainly linked to late endosomal vesicle trafficking (Antonin et al., 2000b) and macroautophagosomal maturation (Chou et al., 2021) and different interaction partners have been identified, including syntaxin 7, syntaxin 8, and endobrevin/VAMP-8 (Antonin et al., 2002). In immune cells, Vti1b has been reported to play a role in lytic granule exocytosis in cytotoxic T cells (Dressel et al., 2010). In addition, it has recently been suggested that Vti1b interacts with the invariant chain of the major histocompatibility complex II (MHCII) and directs the specific membrane fusion events in the early endosomal pathway that allow efficient antigen processing and antigen loading on MHCII (Margiotta et al., 2021). However, no studies have been carried out on Vti1b in B cells.

Here, we investigated the localisation and function of Vti1b in B lymphocytes using cell lines and primary cells from a knock-out (KO) mouse model. We found that Vti1b was mainly localised to the Golgi apparatus and a subset of lysosomes in resting B cells. Upon BCR activation, Vti1b colocalised with the internalised antigen-BCR complexes and it was polarised to the immune synapse. With the hypothesis that Vti1b could play a role in BCR signalling or the vesicular antigen processing pathways, we interrogated the function of Vti1b in B cells using a Vti1b KO mouse. However, we detected no marked differences in these aspects of B cell activation, suggesting redundancy between Vti1b and other SNAREs.

## Materials and methods

### Cells and mice

A20 D1.3 B cells (Williams et al., 1994) were a gift from Prof Facundo Batista (The Ragon Institute of MGH, MIT and Harvard, United States). Cells were maintained in complete RPMI [cRPMI; RPMI 1640 with 2.05 mm L-glutamine, 10% fetal calf serum (FCS), 50 μm β-mercaptoethanol, 4 mm L-glutamine, 10 mm HEPES and 100 U/ml penicillin/streptomycin].

The Vti1b^−/−^ (KO) mouse strain has been previously described (Atlashkin et al., 2003). OT-II mice (Barnden et al., 1998) were a gift from Maija Hollmén (University of Turku, Finland). Wild-type C57BL/6NCrl mice were purchased from the University of Turku Central Animal Laboratory. All strains were on a C57BL/6 background and maintained under specific-pathogen-free conditions. Age- (8–12 weeks), and sex-matched animals and littermate controls (Vti1b^+/-^; HEZ) were used whenever possible. The maintenance of the animals was approved by the Ethical Committee for Animal Experimentation in Finland and adhered to the Finnish Act on Animal Experimentation (62/2006; animal license numbers: 7574/04.10.07/2014 KEK/2014-1407-Mattila, 10727/2018). Maintenance of the Vti1b strain was according to German law and ethical regulations.

### B and T cell isolation

Splenic B cells or T cells were isolated using the EasySepTM Mouse B Cell (#19854, StemCell Technologies) or EasySepTM Mouse T Cell Isolation Kits (#19851, StemCell Technologies) according to the manufacturer’s instructions. Cells were let to recover in cRPMI in an incubator at +37°C and 5% CO_2_ for 1 h before every experiment.

### Transfections

A20 D1.3 cells were transfected as previously described (Šuštar et al., 2018). Cells were resuspended in transfection buffer containing 4 µg of plasmid (GFP-C1 or GFP-Vti1b) and electroporated using AMAXA (program L-013; Biosystem) in 0.2 cm gap electroporation cuvettes. Cells were then transferred to 4 ml of cRPMI to recover overnight. pMRXIP GFP-Vti1b was a gift from Noboru Mizushima (Addgene plasmid #45922).

### Immunofluorescence samples for colocalisation studies

Twelve-well PTFE diagnostic slides (Thermo Fisher, #10028210) were coated with 4 μg/ml fibronectin in PBS. GFP-Vti1b transfected A20 D1.3 cells were resuspended in Imaging Buffer (PBS, 10% FCS) and seeded on the fibronectin-coated wells and incubated at 37°C for 20 min. Then, cells were fixed with 4% PFA for 10 min at RT and blocked/permeabilized for 20 min at RT (5% donkey serum with 0.3% Triton X-100 in PBS). After blocking, samples were stained with primary antibodies (Supplementary Table S1) at 4°C O/N in staining buffer (1% BSA, 0.3% Triton X-100 in PBS), followed by washes with PBS and incubation with the fluorescently-labelled secondary antibodies for 30 min at RT in PBS. Samples were mounted using FluoroMount-G containing DAPI (Thermo Fisher, #00495952). Images were acquired on a 3i CSU-W1 Marianas spinning disk confocal microscope (Intelligent Imaging Innovations) equipped with a ×63 Zeiss Plan-Apochromat objective (NA 1.4) and a Hamamatsu sCMOS Orca Flash4.0 camera (2048 × 2048 pixels, 1 × 1 binning).

### B cell activation and visualization of antigen vesicles by immunofluorescence microscopy

A20 D1.3 were labelled on ice for 10 min with 10 μg/ml of Rhodamine Red X (RRx)-labelled donkey anti-mouse F (ab’)_2_ fragments of anti-IgM antibodies (#715-296-020, Jackson ImmunoResearch), washed with PBS to remove excess unbound antigen and resuspended in Imaging Buffer (PBS, 10% FCS). After washing, cells were seeded on the fibronectin-coated wells and incubated at 37°C to trigger activation (5–60 min). Then, cells were fixed with 4% PFA for 10 min at RT, washed, mounted using FluoroMount-G containing DAPI, and imaged as described above. For live cell imaging, samples were prepared and imaged as described in Hernández-Pérez and Mattila, 2022.

### B cell receptor internalisation

Primary B cells (10^7^/ml) were stained for 10 min on ice with 10 μg/ml of biotinylated anti-IgM (Southern Biotech, #1021-08) in PBS-10% FCS. Labelled cells were washed with PBS, kept on ice, and transferred to a 96-wp (2 × 10^5^/well) for activation at different timepoints (37°C and 5% CO_2_ for 45, 30, 15, and 5 min). As a control (time 0), samples were kept on ice at all times. After incubation, cells were stained with Alexa Fluor^®^ 633 Streptavidin (1:1,000) in PBS on ice for 20 min. Samples were then washed with cold PBS and analysed. A Beckman-Coulter Gallios analyser equipped with three lasers (409, 488, and 633 nm) was used. Data were analysed using FlowJo v10 (Tree Star) as described in Hernández-Pérez and Mattila, 2022, using the geometric mean of the intensity.

### Synapse formation

#### Anti-IgM coated beads

5 µm Streptavidin beads (Bangs Laboratories, # CP01N/10984) were coated with 10 μg/ml of biotinylated goat anti-mouse IgM at 37°C for 30 min (shaking 1,000 rpm) and washed in 2% BSA/PBS. Uncoated beads were used as a negative control. The A20 D1.3 cells transfected with GFP-VTI1b or GFP as control were mixed with the anti-IgM-coated and uncoated beads (1:1) and plated on fibronectin-coated slides. Cells were incubated for 15 and 30 min (+37°C, 5% CO_2_) to induce the activation, fixed in 4% PFA for 10 min at RT and then permeabilized and blocked with 0.3% Triton X-100, 5% donkey serum in PBS, 30 min at RT. To visualize the polarisation of BCRs and actin to the beads, cells were then stained with Donkey anti-mouse IgM F (ab´)_2_ - RRX (Jackson ImmunoResearch, #715-296-020) (1:500) and Alexa Fluor 647 phalloidin (Life Technologies, #A22287) (1:300) for 60 min at RT in staining buffer, washed with PBS, mounted with FluoroMount-G containing DAPI, and imaged as described previously.

#### Anti-IgM coated glass

Twelve-well PTFE diagnostic slides were coated with 10 μg/ml goat anti-mouse IgM F (ab')_2_ (#115-006-020, JIR) in PBS at RT for at least 1 h. Primary B cells were loaded with 1 µM of Cell Trace Violet (CTV) or left unlabelled and mix at a 1:1 ratio (HEZ:KO). GFP-Vti1b or GFP alone transfected A20 D1.3 or mixed primary B cells were seeded on the coverslips and let to get activated for 15 min (+37°C, 5% CO_2_), fixed in 4% PFA for 10 min at RT, and permeabilized/blocked (5% donkey serum with 0.3% Triton X100 in PBS) for 20 min at RT. A20 D1.3 cells were stained with anti-Rab6, Rab7, Rab11, and phalloidin (Supplementary Table S1). Primary cells were stained with Alexa Fluor^®^ 488 anti-phospho-PLCγ2 (1:100; BD Biosciences, 558507) and Acti-Stain 555 Phalloidin (1:150; Cytoskeleton Inc., PHDH1-A) for 1 h in staining buffer. Samples were mounted in FluoroMount-G (Thermo Fisher). Images were acquired on a spinning disk confocal microscope (see above). Cells were visualized at the contact plane and the images of F-actin, and pPLCγ2 were processed with ImageJ using the CTV channel to discriminate between HEZ or Vti1b KO cells. Spreading area (determined on the phalloidin channel) and mean fluorescence intensity of the pPLCγ2 was analysed per cell.

### Western blot

Primary B cells were starved for 20 min (37°C) in plain RPMI, followed by activation with 10 μg/ml of soluble goat anti-mouse F (ab')_2_ IgM (#115-006-020, JIR) for 5, 15, and 30 min. Cells were lysed by adding 4X Laemmli buffer with beta-mercaptoethanol (Bio-Rad) to a final concentration of 1X. Cell lysates were sonicated and boiled (96°C, 5 min). Lysates (450.000 primary cells/25 µl) were run on 10% polyacrylamide gels and transferred to PVDF membranes (Trans-Blot Turbo Transfer System, BioRad). Membranes were blocked with 5% milk in TBST (TBS with 0.05% Tween-20) for 1 h and incubated with primary antibodies in 5% BSA in TBST O/N at 4°C (Sarapulov et al., 2020). Secondary antibody incubations (1:20.000) were done for 1 h at RT in 5% milk in TBST using HRP-conjugated secondary antibodies. Washing steps were done in 10 ml of TBST (5 times, 5 min). Membranes were scanned with ChemiDoc MP Imaging System (Bio-Rad) after the addition of Immobilon Western Chemiluminescent HRP Substrate (WBKLS0500, Millipore).

### Eα peptide presentation

Antigen presentation was measured using the Eα peptide system (Viret and Janeway, 2000). For coating, 200 nm Dragon Green Streptavidin beads (Bangs Laboratories, #CFDG001) were used. Beads were washed with 2% FCS in PBS, sonicated and coated with a 1:1 (w/w) ratio of biotinylated anti-IgM and biotinylated Eα_52-68_ peptide (Biotin-GSGFAKFASFEAQGALANIAVDKA-COOH; Genscript) for 1 h at 37°C. Beads coated with biotinylated anti-IgM alone were used as a control. Cells and beads were incubated for 30 min at 37°C to allow binding and internalisation, washed, and incubated again at 37°C for 4 h. After 4 h, samples were transferred on ice, washed, blocked with Fc-block (Biolegend, #156604) and stained with anti-Eα-MHC-II antibody (Invitrogen, #14-5741-85) for 30 min. Then, samples were washed and stained with Alexa Fluor^®^ 633 goat anti-mouse IgG2b (1:500) for another 30 min. A Beckman-Coulter Gallios analyser equipped with three lasers (409, 488, and 633 nm) was used. Data were analysed using FlowJo v10 (Tree Star).

### Antigen presentation and OT-II proliferation

For coating, 100 nm Streptavidin beads (Bangs Laboratories, #CP01000) were used. Beads were prepared as described for Eα peptide presentation and coated with different ratios of biotinylated anti-IgM and biotinylated ovalbumin (OVA) (produced in-house) for 1 h at 37°C. Beads coated with biotinylated anti-IgM alone were used as a control. B cells purified from WT or KO spleens labelled with 1 µm CFSE and T cells purified from OT-II spleens were labelled with 5 µm Cell Trace Violet (CTV) for 20 min at 37°C in RPMI. Labelled B cells were incubated with the beads for 30 min at 37°C, washed, and incubated with the labelled T cells (1:1) in a 96-well U-bottom plate for 72 h. After 3 days, supernatants were transferred to a new plate for cytokine quantification in ELISA (Hernández-Pérez et al., 2020) and cells were washed, blocked with Fc-block and stained with LiveDead APCe780 (1:1,000; Thermo Fisher, #65-0865-14). Samples were washed one more time and a BD LSR Fortessa analyser equipped with four lasers (405, 488, 561, and 640 nm) was used. T cell proliferation was analysed using the Proliferation Module in FlowJo.

### Statistical analysis and illustrations

Statistical significances were calculated using an unpaired Student’s *t*-test assuming a normal distribution of the data unless otherwise stated in the figure legends. Statistical values are denoted as: *p < 0.05, **p < 0.01, ***p < 0.001, ****p < 0.0001. Graphs were created in GraphPad Prism 6/8, and illustrations were created with BioRender. Figure formatting was done on Inkscape 1.0.

## Results

### Vti1b localises to the Golgi apparatus, plasma membrane and endosomes in B cells

**FIGURE 1:**
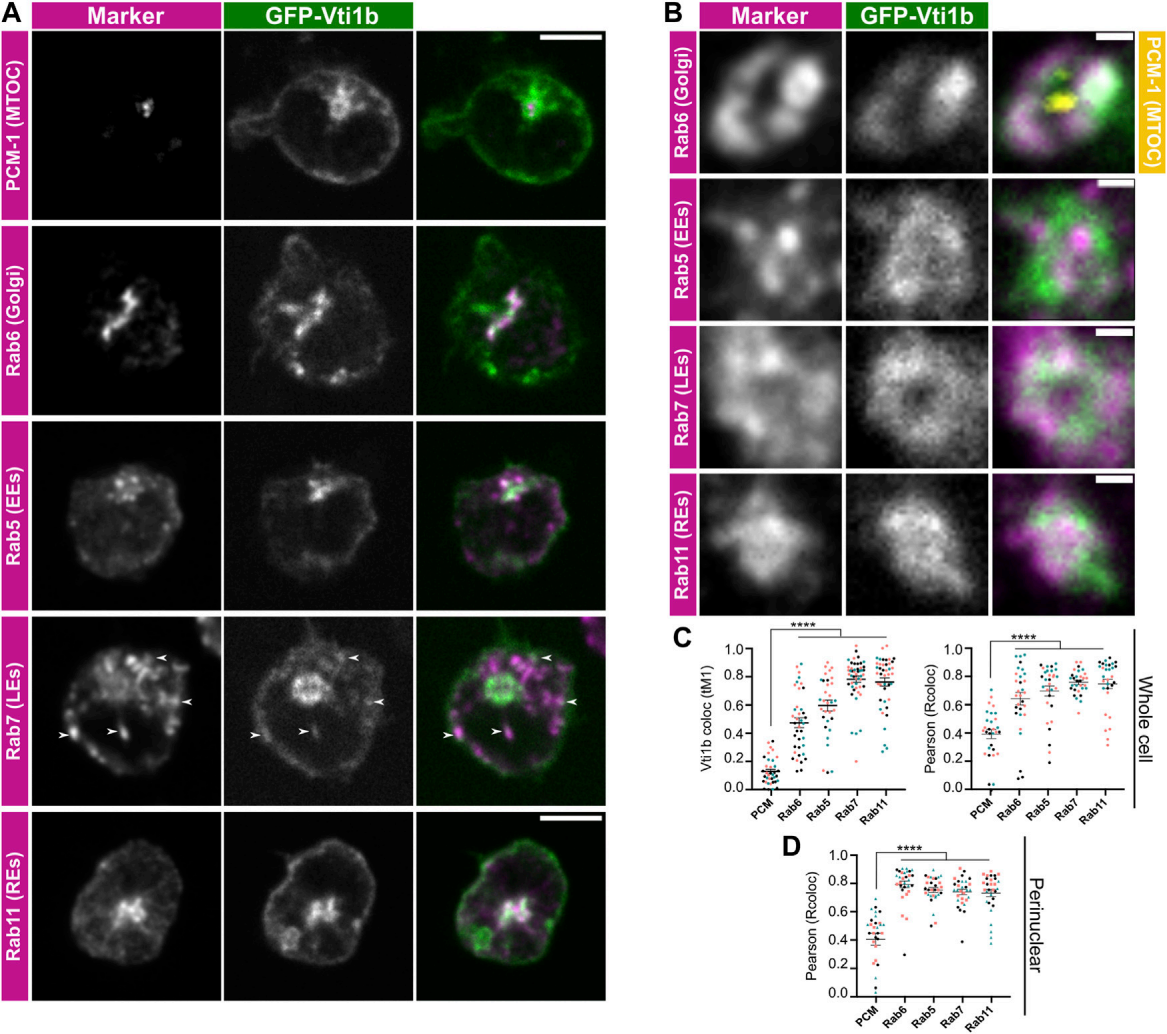
Vti1b is localised to the Golgi complex and endosomes in B cells. **(A,B)** A20 D1.3 B cells were transfected with GFP-Vti1b (in green) and subjected to immunofluorescence analysis (in magenta) with anti-PCM-1 (MTOC), Rab6 (Golgi), Rab5 (early endosomes; EEs), Rab7 (late endosomes/lysosomes; LEs), and Rab11 (recycling endosomes; REs) antibodies. The samples were imaged with a spinning disk confocal microscope. Single representative slices are shown. Scale bar: 5 µm. **(B)** Representative zoom-in images of the perinuclear region are shown with PCM-1 is shown in yellow. Scale bar: 500 nm. **(C,D)** Colocalisation analysis of Vti1b with the different markers in the **(C)** whole cell as shown in **(A)** or **(D)** the perinuclear compartment as shown in **(B)**. Representative results of *n* = 3 independent experiments. Results are shown as mean ± SEM; every dot represents a cell and the colours represent the 3 experiments. Statistics: unpaired *t*-test.

Inspired by the suggested enrichment of Vti1b to the sites of BCR signalling (Awoniyi et al., 2020), we hypothesized that Vti1b could be involved in BCR or antigen trafficking. Hence, we first investigated the localisation of Vti1b in A20 D1.3 cells, a mouse B cell line that expresses a transgenic IgM BCR. To study the endogenous localisation of Vti1b in B cells, we first attempted to validate several commercial antibodies against Vti1b (data not shown) utilizing GFP-Vti1b transfected A20 D1.3 cells as a positive control for the antibody validation. We found only one antibody that recognized the GFP-fused Vti1b in A20 D1.3 cells overexpressing this construct (Supplementary Figure S1A). This antibody also worked for the detection of endogenous human (Raji) and mouse (A20 D1.3) Vti1b (Supplementary Figure S1B). However, the antibody staining still gave a high background signal, challenging the detection and, thus, we deemed the antibodies not suitable to report on the subcellular localization of endogenous Vti1b in our system. Thus, we evaluated the colocalisation of GFP-Vti1b (Supplementary Figure S1C) with different intracellular compartments in transfected A20 D1.3 cells. First, we verified that the transfection did not affect the surface expression of the BCR and MHCII molecules, indicating that the overexpression of Vti1b did not significantly influence membrane trafficking or recycling of these receptors critical for B cell function (Supplementary Figure S1D). Vti1b has been mostly reported to localise to intracellular vesicles, but we also detected localization on the plasma membrane, particularly visible in cells with higher GFP-Vti1b expression (Figures 1A,B). To verify that the plasma membrane localization was not simply a consequence of the overexpression, we analysed the membrane localisation in cells with different levels of overexpression. Although there was a slight trend suggested by the slope of the fitting line, this correlation was rather weak suggesting that high expression levels do not explain the membrane localization (Supplementary Figures S1E–G). Vti1b was also concentrated in some vesicular structures in the cell periphery as well as in the perinuclear area, around the microtubule organising centre (MTOC) (Figures 1A,B). We compared the localization of GFP-Vti1b to different markers and detected perinuclear colocalization with Rab6 (Golgi apparatus), as well as peripheral Rab7^+^ vesicles (late endosomes/lysosomes). Additionally, Vti1b also showed colocalization with the recycling (Rab11^+^) and early endosomes (Rab5^+^) (Figures 1C,D). We concluded that in B lymphocytes, Vti1b is found mainly in the association with the Golgi complex, plasma membrane, and different subsets of endosomes (Rab5^+^, Rab7^+^, and Rab11^+^).

### Vti1b is enriched in the B cell receptor-antigen vesicles after B cell receptor activation

**FIGURE 2:**
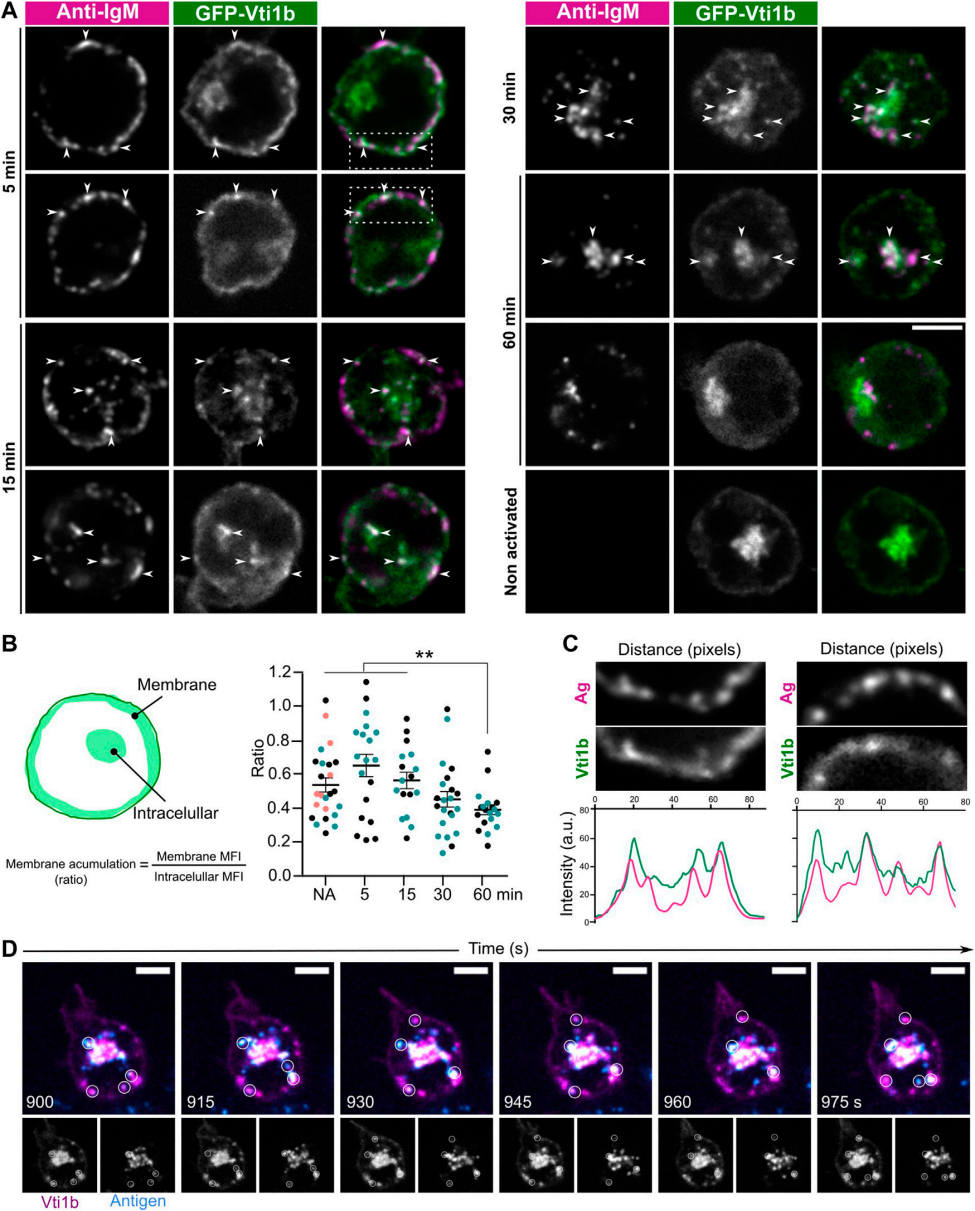
Vti1b partially colocalizes with antigen at the plasma membrane and in the endosomal pathway. **(A)** GFP-Vti1b (green) transfected A20 D1.3 cells were activated (5–60 min) or not (non-activated) with anti-IgM F (ab’)2 fragments as a surrogate antigen (magenta), fixed, and imaged using a spinning disk confocal microscope. Scale bar: 5 µm. Representative images (2D, one plane) are shown (*n* = 2–3 independent experiments). **(B)** Non-activated (NA) or activated (5–60 min) GFP-Vti1b transfected cells were analysed using Fiji ImageJ. The MFI of GFP-Vti1b (membrane and intracellular; see schematics) was obtained and the ratio was calculated to analyse the membrane enrichment of Vti1b before and after activation. Every dot represents one cell; each colour represents one independent experiment (*n* = 2–3 experiments). **(C)** Plot profile analysis showing colocalisation of the surrogate antigen anti-IgM (Ag) and Vti1b on the plasma membrane. The inset corresponds to the dotted square in image 2A. **(D)** Live imaging of Vti1b-GFP (magenta) vesicles together with internalised antigen vesicles (cyan). Frames from Supplementary Movie S1. One frame has been selected every 15 s. The white circles show examples of colocalisation. Scale bar: 5 μm.

In our APEX2 screen (Awoniyi et al., 2020), Vti1b was found to get enriched to the sites of BCR activation, showing a peak at 15 min after BCR activation. Hence, to answer our question if Vti1b responded to BCR activation and was potentially involved in the subsequent membrane trafficking events, we activated the cells with fluorescently labelled anti-IgM F (ab’)_2_ fragments as a surrogate antigen for different time points (Figure 2A). When looking at overall change in Vti1b subcellular localization, we detected increased enrichment of Vti1b to the perinuclear area after 60 min of activation (Figure 2B). We also detected a trend of increase membrane enrichment at 15 min after activation as compared to the resting state but this was not statistically significant. We followed the localisation of Vti1b in more detail and observed that Vti1b was found at the BCR-enriched plasma membrane areas and at sites of antigen-BCR internalisation or internalized antigen vesicles, both visible as clusters close to the plasma membrane (Hernández-Pérez and Mattila, 2022) already at 5 min after activation (Figure 2A, arrows; Figure 2C). Colocalisation of Vti1b with the antigen was also observed at 15 and 30 min, both close to the plasma membrane and in vesicles inside the cell. These data are consistent both with our finding above that Vti1b localizes to the Rab7^+^ vesicles and with our previous studies showing that antigen colocalises with peripheral Rab7^+^ endosomes very soon after internalisation (Hernández-Pérez et al., 2020). We further verified the colocalization of Vti1b and antigen by live cell imaging, where we detected antigen vesicles that clearly moved together with Vti1b-GFP (Supplementary Movie S1). However, Vti1b recruitment to these vesicles was partial, as not all clusters/vesicles colocalised with Vti1b (Figure 2D; Supplementary Movie S1). At later time points, Vti1b showed partial colocalisation with the perinuclear antigen compartments, probably in Rab7^+^ vesicles, but excluded from the Golgi apparatus (Hernández-Pérez et al., 2020).

### Vti1b is recruited to the immune synapse upon activation

**FIGURE 3:**
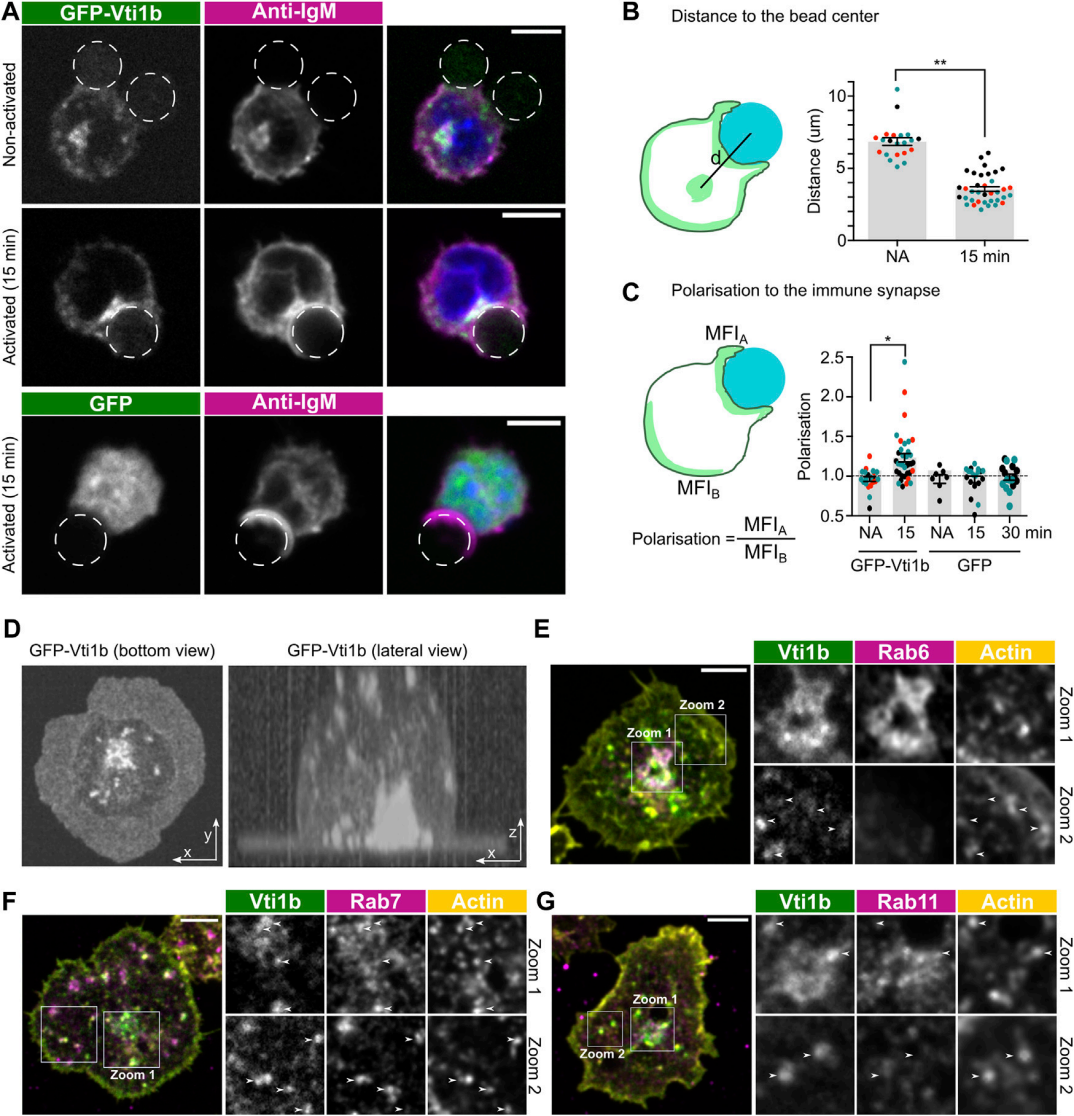
Vti1b is polarised to the IS upon activation. **(A)** A20 D1.3 cells were transfected with GFP-Vti1b or GFP as a control (in green) and activated with anti-IgM coated beads or incubated with uncoated beads (dashed circles) for 15–30 min. Cells were stained with anti-BCR antibodies (magenta) to show the accumulation of the BCR at the sites of activation. Scale bar: 5 µm. **(B)** Quantification of **(A)**. The distance from the endosomal compartment (ROI manually selected) to the center of the bead was quantified as shown in the schematics. NA = non activated. Data are presented as mean ± SEM. Every dot represents one individual cell (*n* = 3 independent experiments, shown in black, red, and green). p** < 0.01. **(C)** Quantification of **(A)**. The polarisation of Vti1b to the IS was quantified as shown in the schematics. NA = non activated. Data are presented as mean ± SEM. Every dot represents one individual cell (*n* = 2–3 independent experiments, shown in black, red, and green). p* < 0.05.**(D)** A20 D1.3 cells were transfected with GFP-Vti1b and seeded on antigen (activated; anti-IgM) coated glass. Left: bottom view. Right: lateral view. **(E–G)** A20 D1.3 cells transfected with GFP-Vti1b (green) were seeded on anti-IgM coated glass for 30 min and stained with **(E)** anti-Rab6 **(F)** Rab7 or **(G)** Rab11 (magenta) and phalloidin (actin; yellow). Scale bar: 5 µm. Arrows show colocalisation of Vti1b with actin foci. Individual channels for the whole cell can be found in Supplementary Figure S2.

*In vivo,* B cells encounter different types of antigens and specific B cells are equipped to efficiently react to antigens in different contexts. Proteinaceous antigens are found in solution or bound to the surface of antigen-presenting cells that express receptors for immunoglobulins and complement decorating the antigens. Activation by the surface-bound antigen triggers formation of an activatory cell-cell interaction structure called immune synapse (IS) (Kuokkanen et al., 2015; Calvo and Izquierdo, 2021). Hence, we next investigated the localisation of Vti1b in the IS triggered by the recognition of surface-bound antigen. To mimic the IS *in vitro*, we activated the B cells using beads or coverslips coated with surrogate antigen. As a control, uncoated beads or fibronectin-coated coverslips were used. First, using the bead system, we observed specific recruitment of GFP-Vti1b to the IS upon BCR activation (Figure 3A). Quantification of the distance of the Golgi-associated, perinuclear Vti1b^+^ compartment and the bead showed a clear polarization of this Vti1b compartment towards IS (Figure 3B). We also quantified the polarization of the membrane-proximal Vti1b signal towards on the bead:cell contact area by comparing the mean fluorescence intensities in the contact site and the distal pole of the cell, and also saw a significant enrichment of Vti1b towards the activation site (Figure 3C).

Next, we employed antigen-coated coverslips to better visualise the IS plane and further examine the localisation of Vti1b. Consistently with the bead activation, we observed Vti1b localization towards the antigen-coated glass (Figures 3D–G; Supplementary Figure S2). We analysed differential patterns of Vti1b in different regions of the cell. In the perinuclear compartment, as in Figure 1, Vti1b highly colocalised with Rab6, further confirming that the majority of Vti1b is found at the Golgi apparatus (Figure 3E; zoom 1). Partial colocalisation of Vti1b with Rab7^+^ and Rab11^+^ structures was also observed in the perinuclear area (Figures 3F,G; zoom 1). Interestingly however, in the periphery of the cell, Vti1b colocalised with F-actin foci and Rab7^+^ structures, but not with Rab11 and Rab6 (Figures 3E–G; zoom 2). These data, together with the previous data showing colocalisation of Vti1b with the antigen vesicles, suggested that Vti1b might participate in antigen internalisation or processing for peptide-MHCII presentation.

### Vti1b deficiency does not lead to major defects in B cell receptor activation and antigen presentation

**FIGURE 4:**
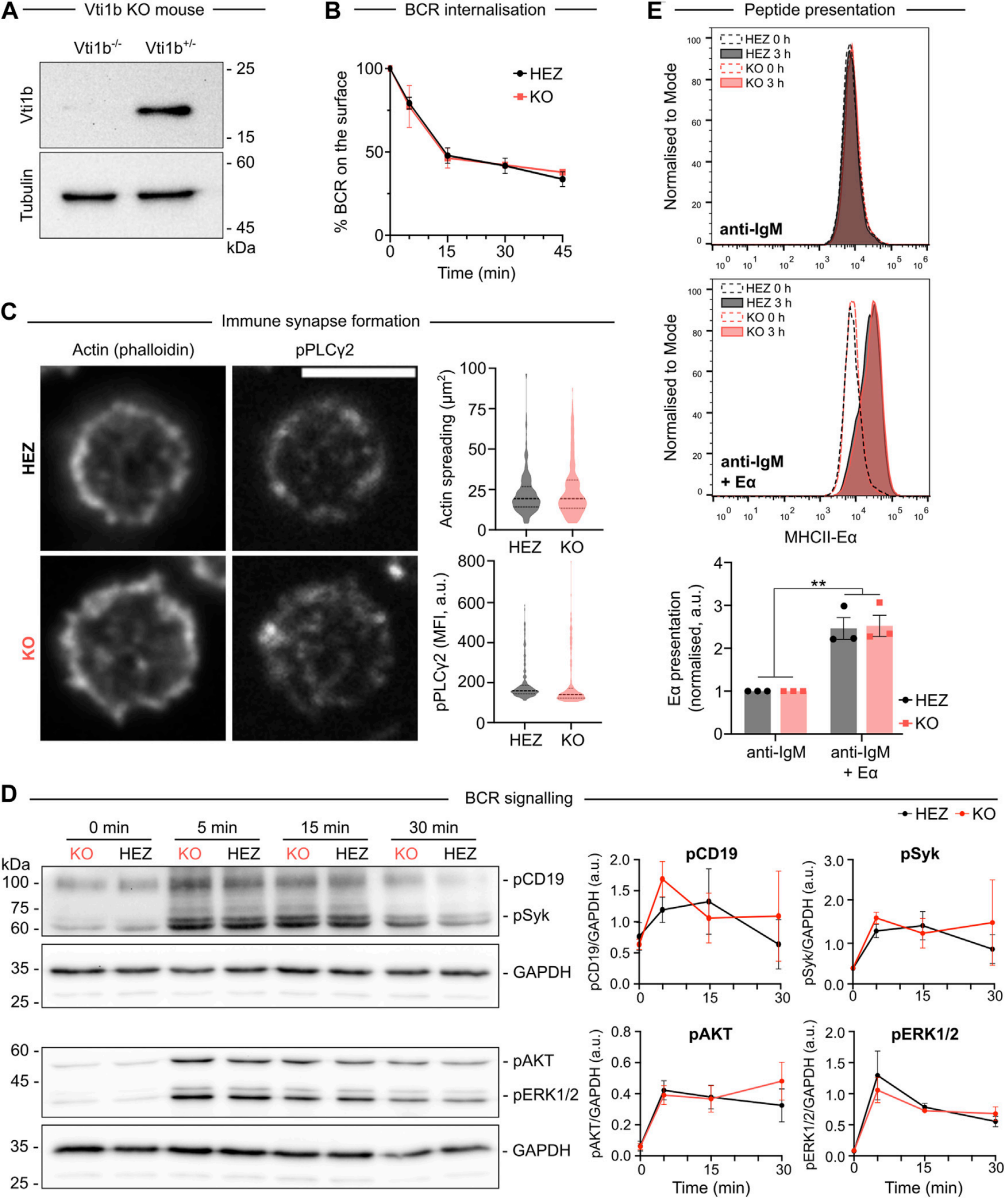
Vti1b-deficiency does not affect antigen internalization, presentation or BCR signalling. **(A)** Western blot showing the expression of Vti1b in Vti1b KO (Vti1b^−/−^) or Vti1b HEZ (Vti1b^+/-^) primary B cells isolated from the spleen. Tubulin was used as a loading control. **(B)** BCR internalisation rate upon antigen [anti-IgM F (ab’)_2_] activation was assessed by flow cytometry in HEZ and KO primary B cells. Results are presented as mean ± SEM (*n* = 3 independent experiments). **(C)** The formation of the immune synapse. Primary B cells were isolated from the spleen and activated on antigen [anti-IgM F (ab’)_2_] coated glass for 15 min. Spreading was quantified based on the F-actin marked area (phalloidin) and signalling based on phospho-PLCg_2_ intensity. Results as presented as violin plots (*n* = 2 experiments). **(D)** Primary B cells were isolated from the spleen of HEZ or KO mice and activated with anti-IgM F (ab’)_2_ fragments for 5, 15, and 30 min. Early (pCD19, pSyk) and late (pAKT, pERK1/2) BCR signalling was analysed using immunoblotting. Results are presented as mean ± SEM (*n* = 3 independent experiments). **(E)** Primary B cells were activated with anti-IgM or anti-IgM + Eα peptide-coated beads and peptide presentation was analysed using antibodies specific for MHCII-Eα peptide complex in flow cytometry. Representative profiles (above) and quantification (*n* = 3 independent experiments; below) are shown. Results are presented as mean ± SEM. p** < 0.01.

Encouraged by our data showing the localisation of Vti1b to the sites of BCR signalling (Awoniyi et al., 2020) and antigen vesicles, we decided to investigate the role of Vti1b using B cells from Vti1b-deficient mice (Atlashkin et al., 2003). We purified primary B cells isolated from the spleen of Vti1b^−/−^ (KO) mice using Vti1b^+/-^ (HEZ) littermates as a control (Figure 4A). First, we evaluated the expression level of IgM BCR on the surface of the cells (Supplementary Figure S3A) and measured BCR internalisation upon activation (Figure 4B) and found no differences. Next, we investigated the impact of Vti1b on BCR signalling and IS formation. Primary B cells were seeded on anti-IgM coated glass and IS formation was analysed by measuring the area spreading based on F-actin intensity, and the BCR signalling based on the intensity of phosphorylated PLCγ2, one of the key downstream components of the BCR signalling. Despite our findings showing polarisation of Vti1b to the IS interface, we did not detect significant defects in IS formation or actin organisation in Vti1b-KO cells (Figure 4C). BCR signalling was further analysed in response to soluble antigens by Western blotting. Primary B cells were stimulated with soluble antigen for different time points to analyse the early (pCD19, pSyk) and late (pAKT, pERK1/2) BCR signalling pathway components, but no significant differences were found (Figure 4D).

As previous studies have suggested that Vti1b interacts with the invariant chain of MHC II to allow antigen processing (Margiotta et al., 2021), we finally analysed antigen processing and presentation in these cells. To do so, we employed the Eα peptide system (Viret and Janeway, 2000), where B cells are activated with beads coated with anti-IgM and Eα peptide. After internalisation, Eα-peptides are loaded onto MHC II molecules and presented on the cell surface, where they can be specifically recognised using antibodies. To our surprise, we detected no differences in the amount of Eα peptide presented on the surface of the cells after 4 h (Figure 4E), suggesting that Vti1b does not play major role in the control of peptide loading and presentation in B cells. To verify this finding using another system, we also tested the antigen presentation using the OT-II system. In this system, B cells are activated with beads coated with anti-IgM and ovalbumin (OVA) and the presented OVA peptides on MHCII are specifically recognised by OT-II T cells that in response proliferate and secrete cytokines. We analysed T cell proliferation upon activation as well as the amount of secreted IL-2 but we found no differences between the WT, HEZ and KO cells (Supplementary Figure S3B and data not shown), further supporting our conclusions. Thus, Vti1b appears largely dispensable for antigen processing and presentation in B cells as no significant defects were detected in Vti1b-KO cells.

## Discussion

B lymphocytes require a fine regulation of the vesicular pathways as endosomal trafficking is involved, among other things, in BCR-antigen internalisation, antigen processing, and presentation, cytokine secretion and antibody production. In our previous proteomic study, we identified the SNARE protein Vti1b among the proteins showing the highest translocation towards the site of BCR activation sites upon antigen encounter (Awoniyi et al., 2020). Although SNARE proteins are critical for membrane fusion events in the cell (Koike and Jahn, 2022), the role of SNAREs, and in particular Vti1b, in B cell biology, remains to be explored. In this work, we found that, despite of enriched localization of Vti1b towards the site of BCR activation and in antigen-containing vesicles, Vti1b-deficient B cells are still able to respond normally to antigen stimulus and process antigen for presentation, suggesting functional redundancy with other SNAREs.

We first investigated the localisation of GFP-Vti1b in B lymphocytes. In T lymphocytes, Vti1b has been found to localise to the perinuclear compartment and close to the plasma membrane (Qu et al., 2011), but no detailed analysis has been reported. In fibroblasts, Vti1b associates with membranes and has been reported to localise to the perinuclear region, but also to endosomes and lysosomes extending into the periphery of the cell (Advani et al., 1998; Antonin et al., 2000b; Kreykenbohm et al., 2002). In B cells, we found Vti1b in the plasma membrane, the Golgi complex, and different endosomal populations positive for Rab5, Rab7, and Rab11 (Figure 1). The widespread localisation of Vti1b could reflect the challenges in the analysis as conventional microscopy cannot properly resolve small structures, such as vesicles under 200 nm, in the crowded cytoplasm of B cells (Hernández-Pérez et al., 2020). Unfortunately, our attempts to image the samples using super-resolution techniques, which require a bright fluorescence signal, were hampered by the lack of functional antibodies and the poor transfection efficiency of the Vti1b plasmid. When studying the localisation of Vti1b upon BCR activation with soluble antigen, we found higher levels of Vti1b at the plasma membrane and peripheral vesicles during the first 15 min after activation as compared to later stages, indicating a shift from higher plasma membrane-proximity to the perinuclear preference during cell activation (Figures 2A,B). Similarly to Rab7^+^ vesicles (see Figure 1), we also detected Vti1b enrichment in a subset of antigen vesicles (Figures 2A,B; Supplementary Movie S1), fitting well with our previous notions that internalised antigen traffics to Rab7^+^ vesicles rapidly after internalisation (Hernández-Pérez et al., 2020) and pointed towards a dynamic function along the antigen processing pathway. At later time points, when antigen clustered in the perinuclear processing vesicles, there was only partial localization with Vti1b, consistent with Vti1b enrichment at the Golgi complex, where the antigen is excluded (Hernández-Pérez et al., 2020).

*In vivo*, B cells recognise antigens not only in solution but also displayed on the surface of antigen-presenting cells (Harwood and Batista, 2010a; Kuokkanen et al., 2015). The recognition of presented antigen leads to the formation of the immune synapse, an important platform for BCR activation and antigen extraction. We observed that Vti1b was specifically polarised to the IS upon BCR activation (Figure 3). It has been widely reported that lymphocytes polarise the endocytic/exocytic machinery, as well as other organelles, such as the Golgi apparatus, to the IS (del Valle Batalla et al., 2018; Duchez et al., 2011; Ulloa et al., 2022; Yuseff et al., 2011). Thus, it was not unexpected that Vti1b, which mainly resides in the Golgi and lysosomes, would polarise to the IS. Interestingly, Vti1b was found also to colocalise with lysosomes and F-actin foci at the IS. It has been proposed that endocytosis and exocytosis occur at these actin foci (Carisey et al., 2018; Roper et al., 2019; Calvo and Izquierdo, 2021), raising a hypothesis that Vti1b could play a role in antigen extraction at these structures.

Next, we interrogated the function of Vti1b in B cells using a Vti1b KO mouse. A previous report showed that Vti1b interacts with the invariant chain responsible for MHCII maturation (Margiotta et al., 2021), and as B cells process the internalized antigen into peptides for presentation on MHCII to gain the critical second signals from cognate T helper cells. We postulated that Vti1b could play a role in endosomal antigen processing pathways or MHCII loading. However, we found no detectable differences in BCR signalling, IS formation, antigen internalisation or antigen presentation in these cells (Figure 4). Vti1b and Vti1a are homologous proteins that are suggested to possess redundant functions. Single gene Vti1b^−/−^ mice and Vti1a^−/−^ mice are viable and fertile, but the double-deficient Vti1a/b^−/−^ mice die at birth (Kunwar et al., 2011), strongly suggesting compensatory roles between the two Vti1 isoforms. Vti1b has also been found to interact with partners of Vti1a (Emperador-Melero et al., 2018; Emperador-Melero et al., 2019). Thus, it would be interesting to investigate the function of the Vti1 SNARE proteins using double-knockout cells in the future. Furthermore, SNARE proteins in general have been reported to possess notable redundancy in their functions and promiscuity in their interaction partners (Koike and Jahn, 2022). In this light, not only Vti1a but also other SNAREs, such as SNAP-29, SNAP-23 or SNAP-47, could partially or totally compensate for the loss of Vti1b (Von Mollard et al., 1997) and to gain a larger picture of SNARE protein function in lymphocyte activation is likely to be a complex task.

## Data Availability

The raw data supporting the conclusion of this article will be made available by the authors, upon request, without undue reservation.

## Supplementary material

SUPPLEMENTARY FIGURE S1
**(A)** A20 D1.3 B cells were transfected with human GFP-Vti1b (green) to test the specificity of the Vti1b antibodies (magenta; Proteintech). Scale bar: 5 µm. **(B)** Non-transfected Raji D1.3 (human) or A20 D1.3 (mouse) B cells were stained with anti-Vti1b antibodies (Proteintech) to detect the endogenous protein. Scale bar: 5 µm. **(C)** A20 D1.3 cells were transfected with GFP or GFP-Vti1b and analysed by flow cytometry. The percentage of transfected cells (GFP+) is shown. **(D)** Untransfected or transfected A20 D1.3 were stained with anti-IgM (anti-BCR) or anti-MHCII antibodies and analysed by flow cytometry. The geometric mean is shown. Every dot represents one measurement. **(E)** Live imaging of GFP-Vti1b transfected A20 D1.3s to better visualise the plasma membrane structures. Cells were transfected, seeded on MatTek dishes and imaged using a spinning disk confocal microscope without PFA fixation. Left: plane. Right: 3D stack reconstruction. **(F)** A20 D1.3 B cells were transfected with human GFP-Vti1b (green) and stained with WGA-AF633 (magenta) to check the plasma membrane localisation. Scale bar: 5 µm. **(G)** GFP-Vti1b transfected cells were analysed using Fiji ImageJ. The MFI (total, membrane and intracellular; see schematics) was obtained and the total intensity was plotted against the membrane accumulation (ratio membrane/intracellular) to analyse the potential correlation between overexpression (total MFI) and membrane localisation of Vti1b. Every dot represents one cell.

SUPPLEMENTARY FIGURE S2 Supplement to Figures 3E–G. A20 D1.3 cells transfected with GFP-Vti1b (green) were seeded on anti-IgM coated glass for 30 min and stained with anti-Rab6, Rab7 or Rab11 (magenta) and phalloidin (actin; yellow). Scale bar: 5 µm.

SUPPLEMENTARY FIGURE S3
**(A)** Surface levels of anti-IgM BCR in non-stimulated primary cells isolated from the spleen of Vti1b HEZ or KO mice. **(B)** Cell Trace Violet (CTV) labelled OT-II T cells were incubated with WT, HEZ or Vti1b KO B cells activated with anti-IgM or anti-IgM:OVA (2 different ratios) coated beads for 3 days. After 3 days, T cell proliferation in response to OVA peptide presentation is measured by flow cytometry.

SUPPLEMENTARY MOVIE S1 A20 D1.3 cells transfected with GFP-Vti1b were pre-labelled with anti-IgM-FIP-647 antibodies (“antigen”) on ice and seeded on poly-L-lysine coated MatTek dishes. After 10–15 minutes incubation at 37°C to allow activation and internalisation of the antigen, a quencher probe was added and cells were imaged using a spinning disk confocal microscope (EVOLVE camera). Magenta: Vti1b-GFP. Cyan: internalised antigen. Merge. One plane was acquired every 5 s. Scale bar: 5 µm.

SUPPLEMENTARY TABLE S1 List of antibodies used in the study.
