# Supplementary material for: The SNARE protein Vti1b is recruited to the sites of BCR activation but is redundant for antigen internalisation, processing and presentation
Source: Front Cell Dev Biol. 2022 Aug 30;10:987148. doi: 10.3389/fcell.2022.987148 (PMC9468668; doi:10.3389/fcell.2022.987148)
Supplement: Supplementary file 1 [file Presentation1.pdf]

The SNARE protein Vti1b is recruited to the sites of BCR activation but redundant for antigen internalisation, processing and presentation

Running title: **Vti1b in B cell activation**

Amna Music[1,2,3*], Blanca Tejeda-González[1,2,3*], Diogo M. Cunha[1,2,3], Gabriele Fischer von Mollard[4], Sara Hernández-Pérez[1,2,3#], and Pieta K. Mattila[1,2,3#]

[1] Institute of Biomedicine, and MediCity Research Laboratories, University of Turku, Finland

[2] Turku Bioscience, University of Turku and Åbo Akademi University, Turku, Finland

[3] InFLAMES Research Flagship Center, University of Turku

[4] Fakultät für Chemie, Biochemie III, Universität Bielefeld, Germany

* Equal contribution

# Shared corresponding authors

Corresponding authors:

Pieta Mattila

E-mail: pieta.mattila@utu.fi

Sara Hernández-Pérez

E-mail: sarher@utu.fi

Keywords: Adaptive immune system, B cells, intracellular traffic, BCR, signalling, SNARE, Vti1b

Supplementary Information

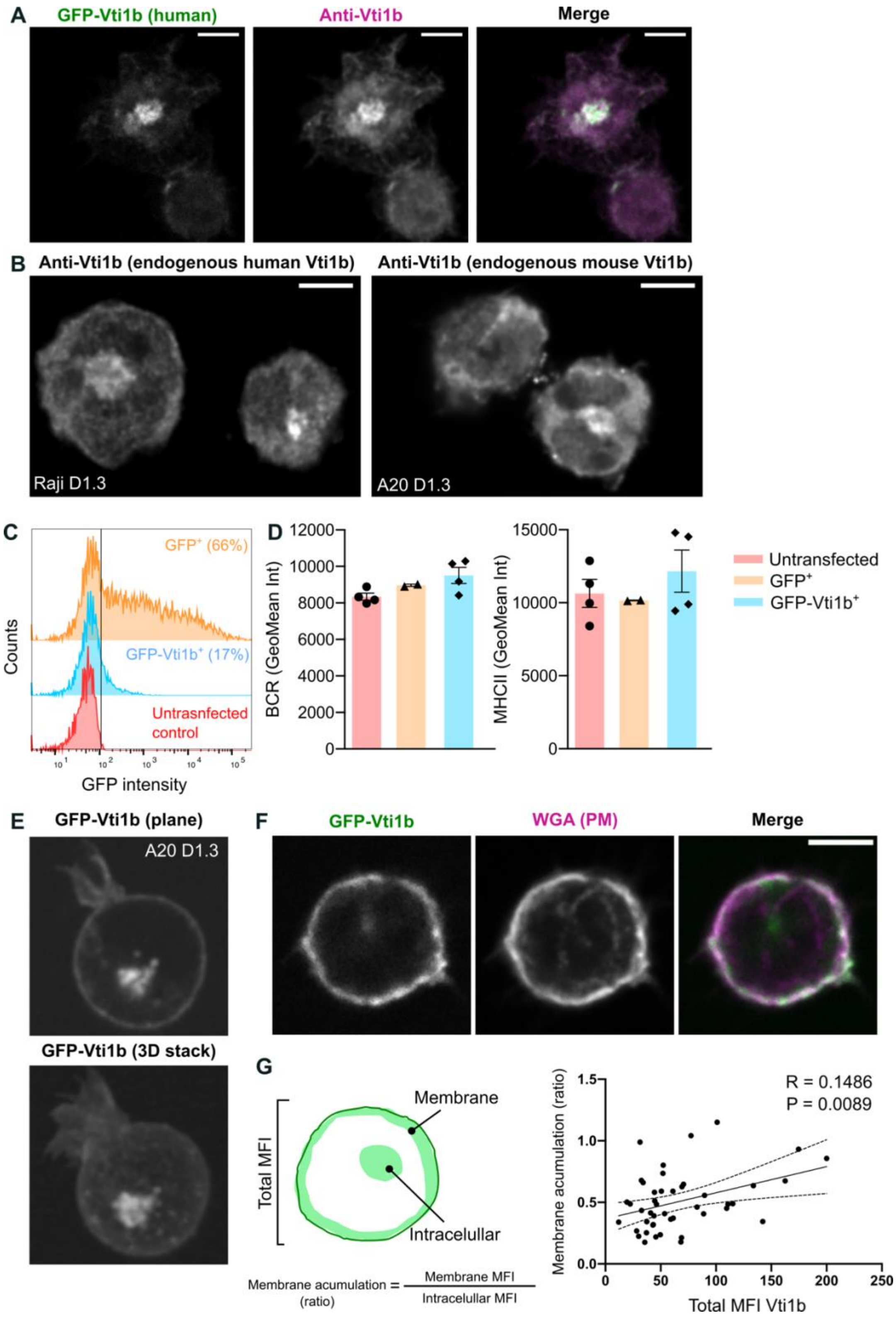

Supplementary Figure 1. (A) A20 D1.3 B cells were transfected with human GFP-Vti1b (green) to test the specificity of the Vti1b antibodies (magenta; Proteintech). Scale bar: 5 µm. **(B)** Non-transfected Raji D1.3 (human) or A20 D1.3 (mouse) B cells were stained with anti-Vti1b antibodies (Proteintech) to detect the endogenous protein. Scale bar: 5 µm. **(C)** A20 D1.3 cells were transfected with GFP or GFP-Vti1b and analysed by flow cytometry. The percentage of transfected cells (GFP+) is shown. **(D)** Untransfected or transfected A20 D1.3 were stained with anti-IgM (anti-BCR) or anti-MHCII antibodies and analysed by flow

cytometry. The geometric mean is shown. Every dot represents one measurement. **(E)** Live imaging of GFP-Vti1b transfected A20 D1.3s to better visualise the plasma membrane structures. Cells were transfected, seeded on MatTek dishes and imaged using a spinning disk confocal microscope without PFA fixation. Left: plane. Right: 3D stack reconstruction. **(F)** A20 D1.3 B cells were transfected with human GFP-Vti1b (green) and stained with WGA-AF633 (magenta) to check the plasma membrane localisation. Scale bar: 5 µm. **(G)** GFP-Vti1b transfected cells were analysed using Fiji ImageJ. The MFI (total, membrane and intracellular; see schematics) was obtained and the total intensity was plotted against the membrane accumulation (ratio membrane/intracellular) to analyse the potential correlation between overexpression (total MFI) and membrane localisation of Vti1b. Every dot represents one cell.

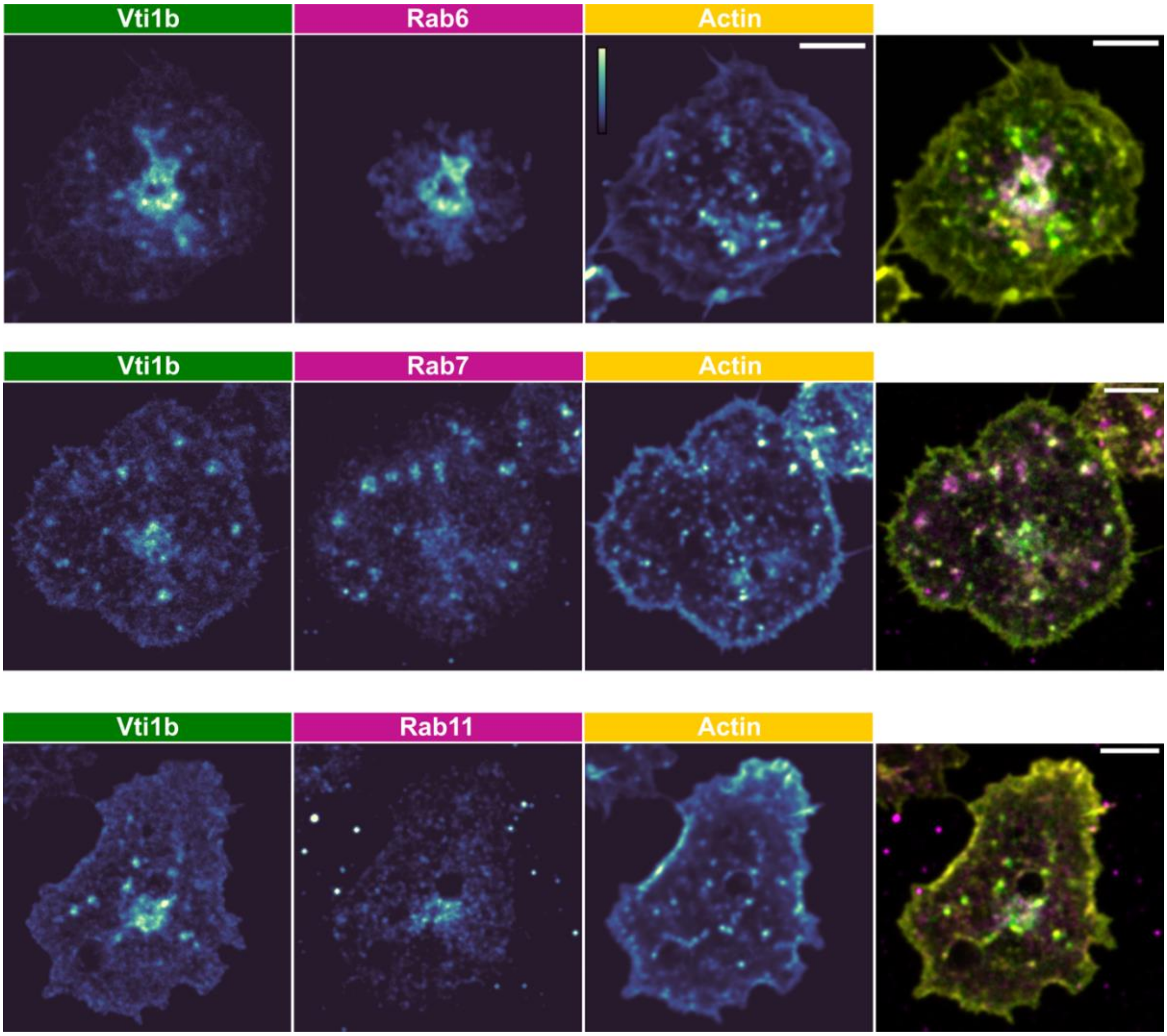

Supplementary Figure 2. Supplement to Figure 3E-G. A20 D1.3 cells transfected with GFP-Vti1b (green) were seeded on anti-IgM coated glass for 30 min and stained with anti-Rab6, Rab7 or Rab11 (magenta) and phalloidin (actin; yellow). Scale bar: 5 µm.

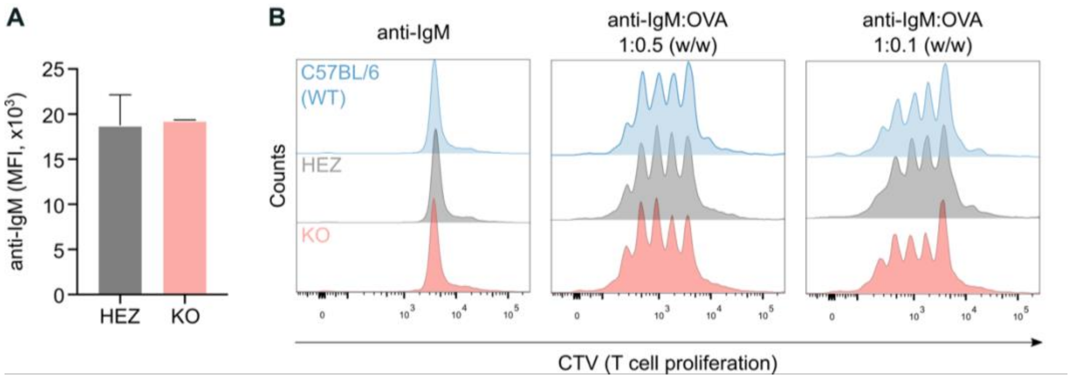

Supplementary Figure 3. (**A**) Surface levels of anti-IgM BCR in non-stimulated primary cells isolated from the spleen of Vti1b HEZ or KO mice. (**B**) Cell Trace Violet (CTV) labelled OT-II T cells were incubated with WT, HEZ or Vti1b KO B cells activated with anti-IgM or anti-IgM:OVA (2 different ratios) coated beads for 3 days. After 3 days, T cell proliferation in response to OVA peptide presentation is measured by flow cytometry.

Supplementary Movie 1. A20 D1.3 cells transfected with GFP-Vti1b were pre-labelled with anti-IgM-FIP-647 antibodies ("antigen") on ice and seeded on poly-L-lysine coated MatTek dishes. After 10-15 minutes incubation at 37 °C to allow activation and internalisation of the antigen, a quencher probe was added and cells were imaged using a spinning disk confocal microscope (EVOLVE camera). Magenta: Vti1b-GFP. Cyan: internalised antigen. Merge. One plane was acquired every 5 seconds. Scale bar: 5 µm.

Supplementary Table 1. List of antibodies used in the study.

Antibody	Company	Clone/Number	Dilution	Technique
Anti-Rab5	Cell Signalling Technologies (CST)	C8B1	1:150	IF
Anti-Rab7	CST	D95F2	1:100	IF
Anti-Rab11	CST	D4F5	1:200	IF
Anti-Rab6	CST	D37C7	1:200	IF
Anti-PCM1-647	Santa Cruz	G-6	1:200	IF
Anti-Vti1b	Proteintech	14495-1	1:100	IF
Donkey anti-Rabbit IgG (H+L) AF555	Thermo	A-31572	1:500	IF
Anti-pCD19	Cell Signalling Technologies (CST)	3571	1:1000	WB
Anti-pSyk	CST	2701	1:1000	WB
Anti-pAKT	CST	4058	1:1000	WB
Anti-pERK1/2	CST	9101	1:1000	WB
Anti-Vti1b	Produced in-house	(Antonin et al., 2000a)	1:1000	WB
Anti-GAPDH	Proteintech	60004-1-Ig	1:10000	WB
Anti-tubulin	Proteintech	66240-1-Ig	1:10000	WB
