# Supplementary figures and images for: The SNARE protein Vti1b is recruited to the sites of BCR activation but is redundant for antigen internalisation, processing and presentation
Source: Front Cell Dev Biol. 2022 Aug 30;10:987148. doi: 10.3389/fcell.2022.987148 (PMC9468668; doi:10.3389/fcell.2022.987148)

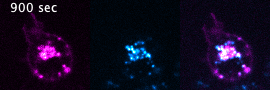

Supplement: Supplementary file 2 [file Image1.TIF]
